# Supplementary material for: The causal relationship between the human gut microbiota and pyogenic arthritis: a Mendelian randomization study
Source: Front Cell Infect Microbiol. 2024 Nov 26;14:1452480. doi: 10.3389/fcimb.2024.1452480 (PMC11629706; doi:10.3389/fcimb.2024.1452480)
Supplement: Supplementary file 1 [file DataSheet1.zip › mendelian test/ebi-a-GCST90027820.csv_forest.pdf]

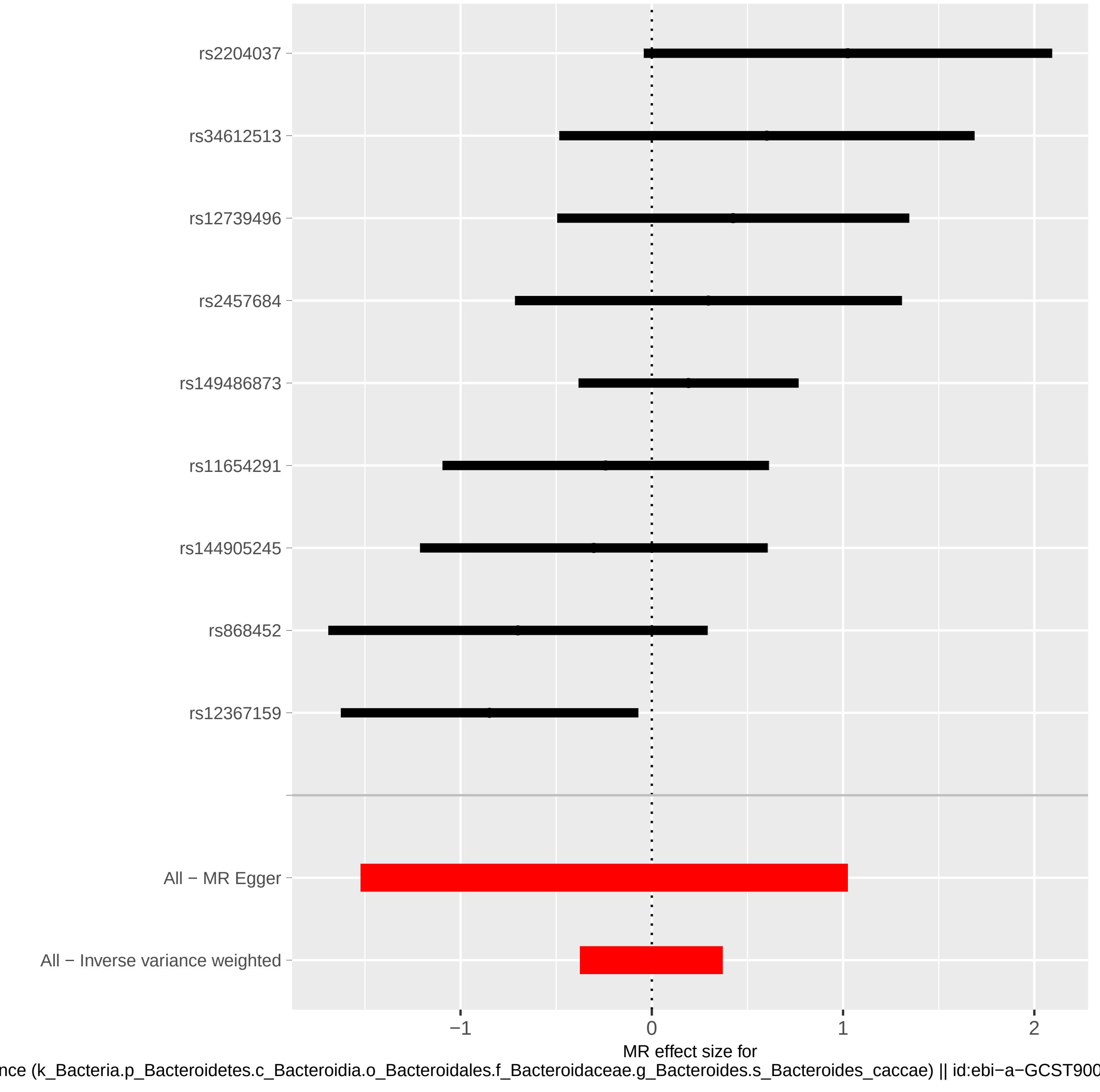

rs2204037
rs34612513
rs12739496
rs2457684
rs149486873
rs11654291
rs144905245
rs868452
rs12367159
All − MR Egger
All − Inverse variance weighted
−1
0
1
2
MR effect size for
nce (k_Bacteria.p_Bacteroidetes.c_Bacteroidia.o_Bacteroidales.f_Bacteroidaceae.g_Bacteroides.s_Bacteroides_caccae) || id:ebi−a−GCST900
